# PDXGEM: patient-derived tumor xenograft-based gene expression model for predicting clinical response to anticancer therapy in cancer patients

**DOI:** 10.1186/s12859-020-03633-z

**Published:** 2020-07-06

**Authors:** Youngchul Kim, Daewon Kim, Biwei Cao, Rodrigo Carvajal, Minjung Kim

**Affiliations:** 1 Department of Biostatistics and Bioinformatics, H. Lee Moffitt Cancer Center and Research Institute, 12902 Magnolia Drive, Tampa, Florida 33612-9416 USA; 2 Department of Gastrointestinal Oncology, Moffitt Cancer Center, Tampa, Florida 33612-9416 USA; 3 Biostatistics and Bioinformatics Shared Resource, H. Lee Moffitt Cancer Center and Research Institute, 12902 Magnolia Drive, Tampa, Florida 33612-9416 USA; 4 Department of Cell Biology, Microbiology and Molecular Biology, University of South Florida, Tampa, FL 33620 USA

**Keywords:** Patient-derived xenograft model, PDX, Gene expression, Predictive cancer biomarker, Chemotherapy, Targeted therapy, Drug response prediction

## Abstract

**Background:**

Cancer is a highly heterogeneous disease with varying responses to anti-cancer drugs. Although several attempts have been made to predict the anti-cancer therapeutic responses, there remains a great need to develop highly accurate prediction models of response to the anti-cancer drugs for clinical applications toward a personalized medicine. Patient derived xenografts (PDXs) are preclinical cancer models in which the tissue or cells from a patient’s tumor are implanted into an immunodeficient or humanized mouse. In the present study, we develop a bioinformatics analysis pipeline to build a predictive gene expression model (GEM) for cancer patients’ drug responses based on gene expression and drug activity data from PDX models.

**Results:**

Drug sensitivity biomarkers were identified by performing an association analysis between gene expression levels and post-treatment tumor volume changes in PDX models. We built a drug response prediction model (called PDXGEM) in a random-forest algorithm by using a subset of the drug sensitvity biomarkers with concordant co-expression patterns between the PDXs and pretreatment cancer patient tumors. We applied the PDXGEM to several cytotoxic chemotherapies as well as targeted therapy agents that are used to treat breast cancer, pancreatic cancer, colorectal cancer, or non-small cell lung cancer. Significantly accurate predictions of PDXGEM for pathological response or survival outcomes were observed in extensive independent validations on multiple cancer patient datasets obtained from retrospective observational studies and prospective clinical trials.

**Conclusion:**

Our results demonstrated the strong potential of using molecular profiles and drug activity data of PDX tumors in developing a clinically translatable predictive cancer biomarkers for cancer patients. The PDXGEM web application is publicly available at http://pdxgem.moffitt.org.

## Background

Cytotoxic chemotherapy and targeted therapy play important roles in the treatment of cancer, alongside with surgery, radiotherapy and a recent breakthrough immunotherapy. Responses of cancer patients to drugs of those anticancer therapies vary widely because of the substantial heterogeneity in the molecular characteristics of their tumors even with a histologically same subtype of cancer [1]. Although a considerable number of novel anticancer drugs have been introduced during the past few decades, overall survival (OS) and quality of life of cancer patients have not been improved much, mainly because of the unselective use of these drugs in the presence of heterogeneous tumor characteristics and drug responses [2]. Hence, it is necessary to develop a personalized anticancer therapy that can help guide individual patients with heterogeneous tumors to anticancer drugs with the most therapeutic benefit. Successful personalized anticancer therapy will then greatly depend on the identification of predictive cancer biomarkers that can be used to accurately select patients who will benefit from treatment with the anticancer drugs [3].

For a predictive cancer biomarker discovery, it is considered most desirable to analyze molecular profiling data and clinical outcome data of cancer patients that were obtained before and/or after a treatment with anticancer drugs of interest from a prospective randomized clinical trial [4]. However, it is not straightforward to develop cancer biomarkers in this manner due to extremely huge cost and time spent in the process of the clinical trial. Because of these limitations, many cancer biomarker studies rely on testing anticancer drugs in preclinical cancer models including immortalized cancer cell lines and animal models [5].

Cancer cell lines cultured in vitro are cancer cells that keep dividing and growing over time, under certain conditions in a laboratory. Human cancer-derived cell lines have been widely used to understand molecular characteristics and drug activity mechanism of tumor cells. For instance, two large cancer cell line panels, Genomics of Drug Sensitivity in Cancer and Cancer Cell Line Encyclopedia, were established to develop new anticancer drugs and to identify new molecular drug targets and predictive biomarkers by interrogating pharmacogenomic mechanisms in more than 1000 cancer cell lines [6, 7]. We and many other research teams have been developing techniques to translate cancer cell line-driven biomarkers into prediction models of cancer patients’ anticancer drug responses [8–13]. Despite these efforts, there still remains a lack of well-validated biomarkers and methods for further biomarker discoveries.

A patient-derived xenograft (PDX) is a promising preclinical model of cancer in which the tissue or cells from a patient’s tumor are implanted into an immunodeficient or humanized mouse. It is used to create an environment that allows for the natural growth of cancer, its monitoring, and the corresponding treatment evaluations of the original patient. Recently, large PDX-based studies, such as National Cancer Institute MicroXeno project, Novartis PDX panel, and EuroPDX consortium study, have interrogated molecular characteristics. These studies, which were based on multiplex molecular platforms including gene expression and genetic mutation, reported that PDXs can retain the distinct characteristics of different tumors from different patients and therefore can effectively recapitulate the intra- and inter-tumor heterogeneity that represents human cancer [14–17]. These novel and unprecedented PDX resources have the potential to provide an opportunity to discover highly predictive cancer biomarkers that can be used to help guide cancer patients to highly beneficial anticancer therapeutics and to accelerate the process of new drug development. However, very few attempts have been made and no analytic tool for developing a PDX-based predictive gene expression model (GEM) is yet available. To address this, we have developed a new pharmacogenomics pipeline, so-called PDXGEM, that can be used to construct a highly predictive GEM of clinical responses of cancer patients to anti-cancer drugs on the basis of pretreatment gene expression profiles and posttreatment drug screening data of the preclinical PDX tumors.

In the present study, we provide a full description of the PDXGEM pipeline and demonstrate its predictive utility by applying it to several cytotoxic and targeted therapeutic agents and validating the prediction performance of resultant multi-gene expression models on independent external cancer patient cohorts with well-annotated clinical outcomes. We have also created a publicly available web-based application with an initial inventory of the data of the Novartis PDX panel and cancer patient cohorts that were used to develop and validate our PDXGEM.

## Results

**Fig. 1:**
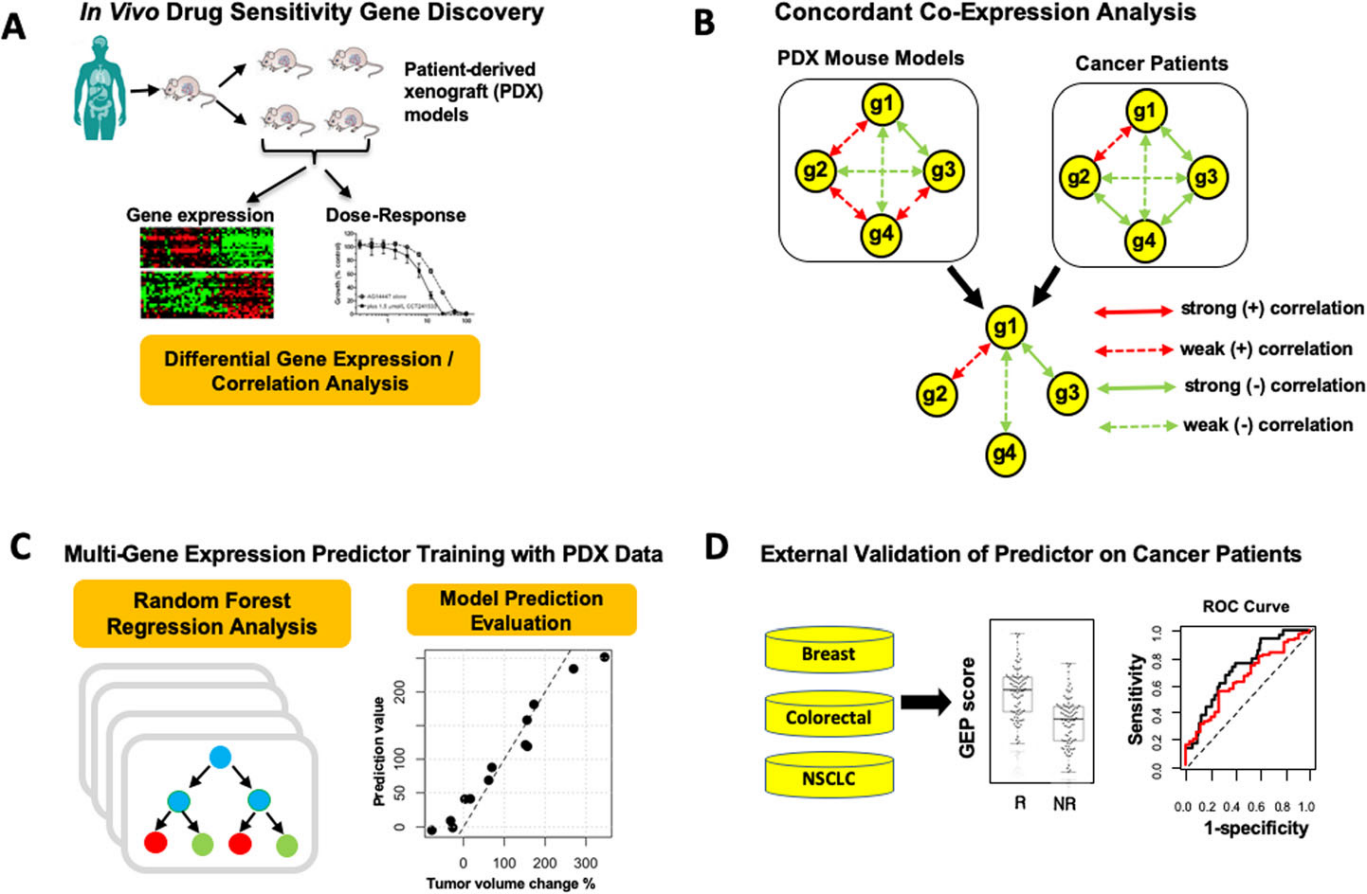
Schema of the patient-derived xenograft based gene expression model (PDXGEM). **a** In the drug sensitivity gene discovery step, correlation analysis and differential expression analysis of gene expression data and drug-activity data in patient-derived xenograft (PDX) tumors are conducted. **b** Concordant co-expression analysis identifies a drug sensitivity gene (g1) that is concordantly co-expressed with 3 other genes (g2, g3, and g4) between PDX tumors and pretreatment cancer patients’ tumors. **c** A multi-gene expression model of drug response is trained on PDX data using the random-forest algorithm. **d** The performance of the multi-gene expression model is validated by contrasting prediction scores between the responsive (R) and the non-responsive (NR) patients to a drug in cancer patient cohort

The PDXGEM pipeline consists of four subsequent steps, 1) drug sensitivity biomarker discovery, 2) concordant co-expression analysis (CCEA), 3) multi-gene expression model training for drug response prediction, and 4) model validation (Fig. 1; see *Materials and Method*). To demonstrate the utility of the PDXGEM, we applied the PDXGEM to building predictive GEMs of cancer patients’ responses to each of three chemotherapy agents and three targeted therapy drugs: paclitaxel and trastuzumab for breast cancer, 5-fluorouracil (5FU) and cetuximab for colorectal cancer (CRC), gemcitabine for pancreatic cancer, and erlotinib for non-small cell lung cancer (NSCLC). External validations of the resultant GEMs were conducted using publicly available gene expression data and clinical outcome data of independent cancer patient cohorts from prospective clinical trials or observational studies.

### PDXGEM for predicting paclitaxel response in breast cancer patients

**Fig. 2:**
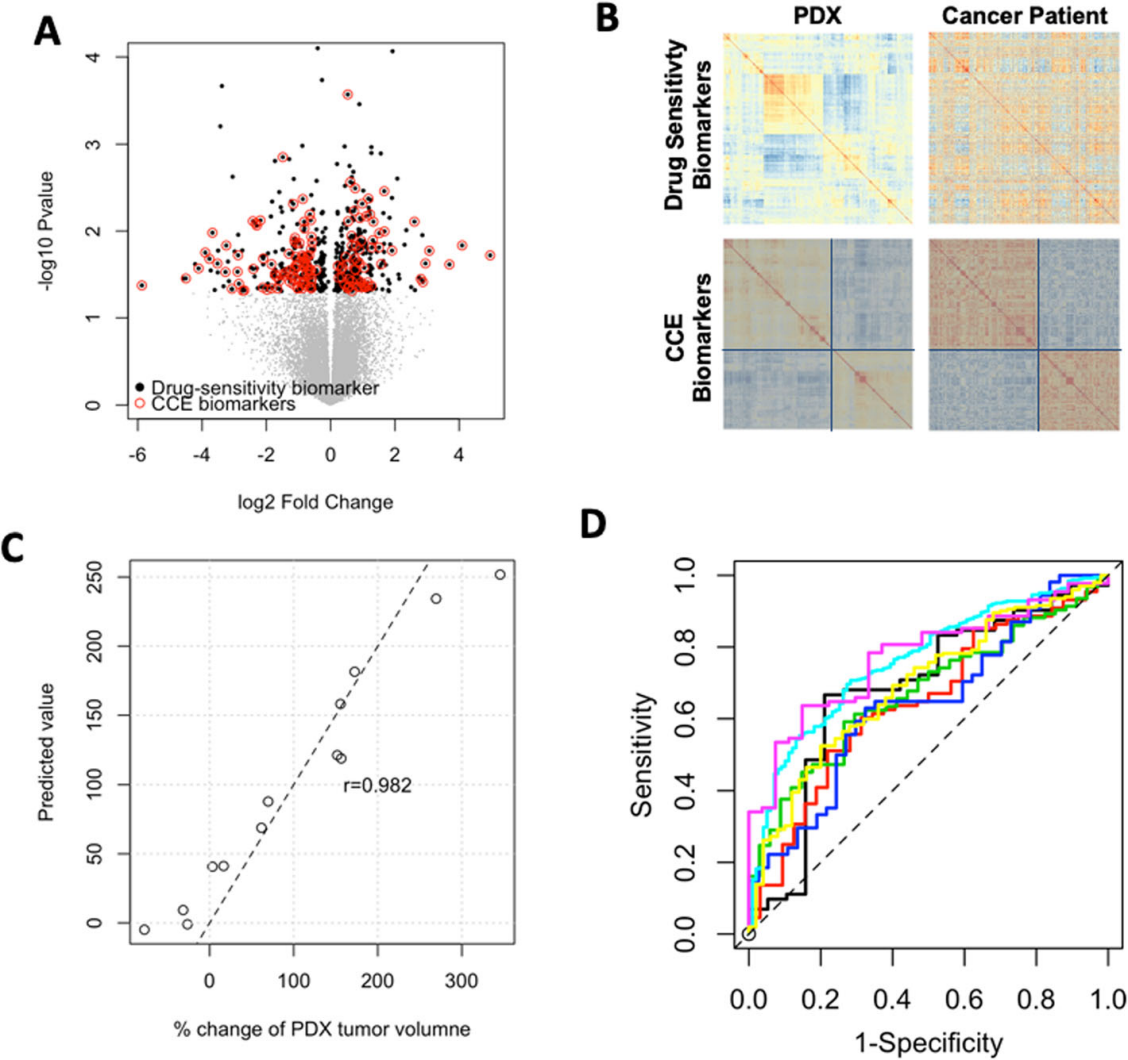
Development of PDXGEM for paclitaxel response prediction in breast cancer patient. **a** Volcano plot with log_2_ fold change of differential gene expressions (x-axis) in paclitaxel-sensitive and paclitaxel-resistant patient-derived xenograft (PDX) models and –log_10_*P*-value (y-axis). Black dots display the initial drug sensitivity probesets and red circles further indicate concordantly co-expressed biomarkers between the PDX models and breast cancer patients. **b** Clustering heatmap depicts correlation matrices of drug sensitivity genes in PDX models (left panel) and pretreatment cancer patients (right panel) before (top panel) and after (bottom panel) concordant co-expression. **c** The Pearson’s correlation coefficient between observed percent change in PDX tumor volumes (x-axis) and PDXGEM prediction scores for breast cancer PDX models (y-axis) was 0.982. **d** Receiver-operating characteristics curves of paclitaxel PDXGEM on seven different breast cancer data sets

Paclitaxel, combined with FAC (fluorouracil, doxorubicin, and cyclophosphamide) is a cornerstone of the current standard chemotherapy used for treating breast cancer patients. We applied PDXGEM to build a multi-gene expression model to predict who may achieve a pathological complete response (pCR) to paclitaxel. Six hundred probesets were first identified as initial drug sensitivity biomarkers that exhibited differential expressions between three breast cancer PDXs with shrunken tumor volumes and ten breast cancer PDXs with increased tumor volumes after receiving paclitaxel (*t*-test nominal *P* < 0.05, Fig. 2a). The pattern of co-expression among the drug sensitivity genes, as measured by a gene-gene correlation coefficient, in the breast cancer PDXs were then quite distinct from that in breast cancer patients (Fig. 2b). This finding is in line with that of a previous study, which showed an inherent biological gap between PDX tumors and their origin cancer patient tumors because of different growth environments surrounding the tumors.

The CCEA showed that concordance co-expression coefficients (CCECs) ranged from − 0.191 to 0.464 for all drug sensitivity biomarkers. Supplementary Figure 1 shows the distribution of all CCECs and scatter plots of gene-gene correlation coefficients for drug sensitivity biomarkers with varying CCEC values. 147 (24.5%) of the drug sensitivity biomarkers showed significantly positive CCECs, ranging from 0.204 to 0.464 between those breast cancer PDXs and a cohort of 251 breast cancer patients (GSE3494 [18]), and we hereafter referred to as the concordant co-expression (CCE) biomarkers. The CCE biomarkers showed more concordant co-expression patterns with two common clusters of genes between the breast cancer PDXs and patients and also had an increased median CCEC of 0.272 (Fig. 2b; bottom) compared with all drug sensitivity biomarkers that did not have common clusters and yielded a median CCEC of 0.09 (Fig. 2b; top).

A random forest (RF) predictor was then trained using the gene expression data of the breast cancer PDXs for all the CCE biomarkers as a model training set. A resultant RF predictor consisted of 145 CCE biomarkers with a positive variable importance value (Supplementary Fig. 2). Prediction scores of the RF predictor, hereafter referred to as PDXGEM score, was tightly correlated with the observed tumor volume changes in the PDX training dataset (*r* = 0.982, *n* = 13; *P* < 0.01; Fig. 2c).

To ensure the predictive performance of the RF predictor, we validated it on seven independent gene expression datasets of breast cancer patients that were collected through four randomized clinical trials (GSE20271 [19], GSE22226 [20], GSE41998 [21], GSE42822 [10]), two prospective observational studies (GSE25065 [22], GSE32646 [23]), and one retrospective study cohort (GSE20194 [24]). Notably, there were significant differences in prediction scores between patients with pCR and those with residual of disease (RD) after paclitaxel-based chemotherapy in all the breast cancer cohorts (*P* < 0.05; Supplementary Fig. 3A-G). In addition, area under the receiver-operating characteristic (ROC) curve (AUC) as an overall classification accuracy ranged from 0.653 to 0.789 (Fig. 2d). To further determine whether the RF predictor is predictive of paclitaxel-specific response, we tested it in 87 breast cancer patients in the GSE20271 clinical trial cohort who did not receive paclitaxel but only FAC combination chemotherapy. There was no significant difference in prediction scores, suggesting that our predictor is predictive of response specifically to paclitaxel (AUC = 0.589, *P* = 0.44; Supplementary Fig. 3H).

To examine the utility of CCEA, we trained a RF predictor using all 600 initial drug sensitivity biomarkers that did not undergo CCEA. Although this predictor was approximately three times complex as the above final RF predictor, there was no significant difference in its prediction scores between pCR and RD groups in four breast cancer cohorts (Supplementary Fig. 4). Furthermore, decreased AUCs were observed in the remaining validation sets, suggesting that the CCEA lead to a parsimonious gene expression signature with a more accurate prediction performance.

Lastly, gene ontology (GO) analyses, which were performed to understand the biological functions of 145 biomarkers of our final paclitaxel response predictor, showed that COL1A1, RPH3AL, and THSD4 were the most significantly associated with breast neoplasm function (false discovery rate (FDR) *P* < 0.001). In addition, DNA replication proteins and mismatch repair were the top two representative pathways (Supplementary Table 1).

### PDXGEM for Trastuzumap-specific response in breast cancer patients

**Fig. 3:**
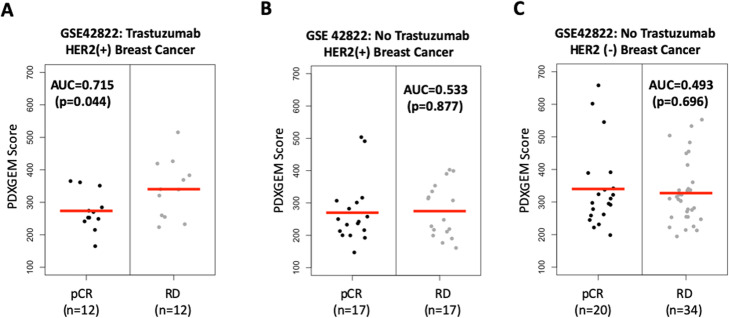
PDXGEM prediction scores for trastuzumab in breast cancer patients by HER2 status. Distributional plot of PDXGEM prediction scores between patients with pathological complete response (pCR) and patients with residual of disease (RD) after receiving trastuzumab (**a**) in HER2 positive breast cancer patients, (**b**) in HER2 positive breast cancer patients who did not receive trastuzumab but did receive other chemotherapy and (**c**) HER2 negative breast cancer patients who did not receive Trastuzumab. Red center lines represent the mean of prediction scores

Trastuzumab is a monoclonal antibody used to treat human epidermal growth factor receptor 2- (HER2-) positive breast cancer by itself or in combination with other anti-cancer therapeutics [25]. To construct a gene signature predictive of response to the trastuzumab in breast cancer patients, we applied the PDXGEM to data on pretreatment gene expression and post-treatment tumor volume changes in 13 breast cancer PDXs that underwent a monotherapy with trastuzumab. We identified 1333 drug sensitivity biomarkers with significant Spearman rank correlation relationships (nominal *P*-value < 0.05) between gene expression levels and the tumor volume changes. We then further screened 515 CCE biomarkers with significant CCECs ranging from 0.201 to 0.509. Finally, an optimal predictor was constructed with 480 CCE biomarkers possessing positive variable importance in RF model training analysis and the predictor yielded a strong correlation coefficient of 0.977 (*p* < 0.01, *n* = 13) between predicted and observed tumor volume changes in the breast cancer PDX models. We then performed an independent validation of this RF predictor using data from the US Oncology 02–103 breast cancer trial (GSE42822 [10]), in which 25 patients with stage II-III HER2-positive breast cancer received trastuzumab. We observed a borderline significant difference in prediction scores between 12 patients with pCR and 13 patients with RD after treatment with trastuzumab (AUC = 0.712, *P* = 0.074). Considering the large number of the biomarkers involved in the predictor and the encouraging AUC value, we set the more stringent threshold value of 0.3 for CCEC at the CCEA step of the PDXGEM pipeline to yield a less complex GEM with more concordantly co-expressed biomarkers between the breast cancer PDXs and patients. As expected, a new RF predictor was constructed with 193 CCE biomarkers and yielded a more significant difference in prediction scores between pCR and RD response groups in the breast cancer trial cohort (AUC = 0.737; *P* = 0.025) (Fig. 3a). To assess the specificity of the RF predictor for trastuzumab, we validated the RF predictor on 34 HER2-positive and 54 HER2-negative breast cancer patients who did not receive trastuzumab in the same clinical trial. In both HER2 strata, we observed no difference in prediction scores between pCR and RD response groups (AUC = 0.533 and *P* = 0.877 for the HER2 positive breast cancer; AUC = 0.493 and *P* = 0.696 for the HER2 negative breast cancer; Fig. 3b). When the predictor was further tested using other available breast cancer patient cohorts treated with paclitaxel-based (not trastuzumab-based) chemotherapy, none of the breast cancer cohorts showed any significant difference in prediction scores, strongly suggesting that the RF predictor is predictive of trastuzumab-specific response in breast cancer patients (Supplementary Fig. 5).

Finally, GO analysis of the 193 biomarkers in the final predictor identified the most significant pathways including miRNA targets in extraceullar matrix and membrane receptors, the focal adhesion-PI3K-Akt-mTOR-signaling pathway, the inflammatory response pathway, and the apoptosis-related network due to altered Notch3 (FDR *P* < 0.05; Supplementary Table 1). In particular, the PI3K-Akt-mTOR-signlaing pathway is a downstream pathway of HER2 and is well known to be responsible for promoting cell proliferation and angiogenesis [26]. In addition, COLTA1 gene had the second highest variable importance in the RF model training analysis and was reported in the genomic study of a phase 3 clinical trial for trastuzumab to be a key gene in integrin signaling pathway which was linked to a decreased recurrence-free survival time after adjuvant trastuzumab therapy [27].

### PDXGEM for predicting response to gemcitabine in pancreatic cancer patients

Gemcitabine is currently used as a backbone in a first-line or second-line treatments for pancreatic ductal adenocarcinoma (PDA), which carries a dismal prognosis with a typical overall survival (OS) of 6 months from diagnosis [28]. Although only six pancreatic cancer PDXs were available for tumor volume changes after receiving gemcitabine treatment in the Novartis PDX panel, we used PDXGEM to develop a gene signature predictive of response to gemcitabine.

We screened 965 drug sensitivity biomarkers using *t*-test to contrast the expression levels of an individual probeset between two PDXs with shrunken tumor volumes and four PDXs with increased tumor volumes after receiving gemcitabine (nominal *P* < 0.05). We further selected 404 CCE biomarkers from CCEA using pretreatment gene expression data of 39 patients with PDA (GSE15471 [29]). In a RF model training analysis of the PDX dataset, the final prediction model consisted of 298 CCE biomarkers. A high correlation coefficient of 0.959 was observed between predicted scores and observed percent changes in PDX tumor volumes.

**Fig. 4:**
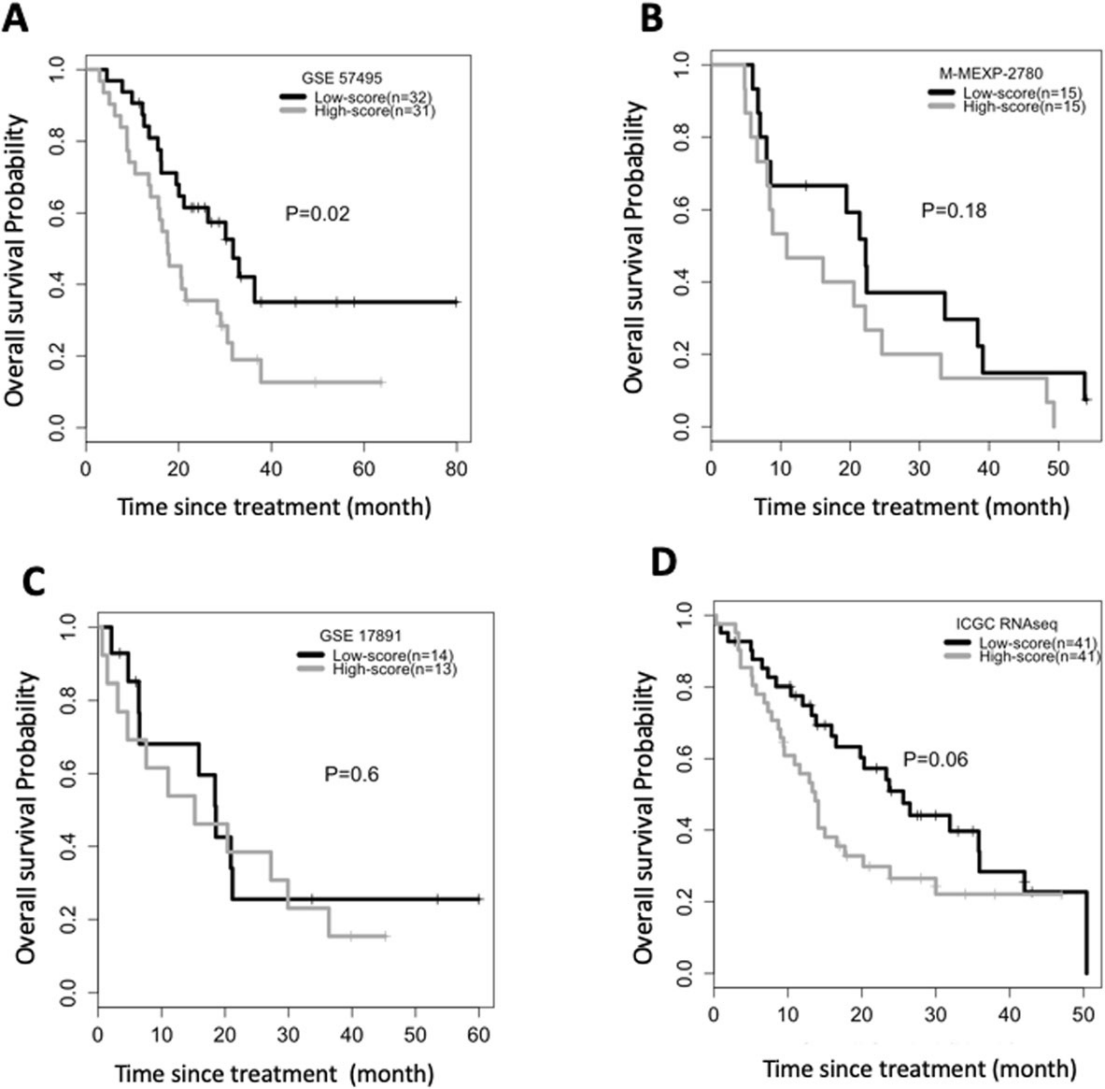
PDXGEM for gemcitabine in pancreatic cancer patients. **a**-**d** Kaplan-Meier curves of overall survival between pancreatic cancer patients with a higher (gray) and lower (black) PDXGEM score than the median prediction score in (**a**) GSE57495, (**b**) M-MEXP-2780, (**c**) GSE17891, and (**d**) ICGC cohort. *P*-value was calculated using log-rank test

As an external validation of the prediction performance of the final model, we collected gene expression data and survival outcome data from a retrospective study cohort of 63 patients with stage I/II PDA who received gemcitabine (GSE57495 [30]). For a comparative analysis of the survival outcomes, we defined two patent subgroups according to whether patients’ prediction scores were higher or lower than the median prediction score. The low-score group then showed a significantly better OS (median OS = 31.7 months, 95% CI = 19.5 ~ not reached) than the high-score group (median OS = 7.7 months, 95% CI = 13.5–28.3, log-rank *P* = 0.023) (Fig. 4a). To assess the prediction ability of the final model for gemcitabine-specific response, we analyzed in a similar manner survival outcome data from a prospective observational study cohort of 30 patients with PDA who did not receive adjuvant chemotherapy (M-MEXP-2780 [31], ArrayExpress). No significant difference was observed in OS, but the low-score group had more promising OS than the high-score group (Fig. 4b; median OS = 22.9 months for the low-score group and 10.9 months for the high-score group; log-rank *P* = 0.18), implying that our PDXGEM signature was predictive of gemcitabine response and partly prognostic. To further confirm the prognostic value of the predictor, we analyzed two additional cohorts of patients with PDA (GSE17891 [32];*n* = 29) and the International Cancer Genome Consortium [33] (ICGC;*n* = 82) even though their chemotherapeutic treatment records were not available. In the GSE17891 cohort, we observed slightly better OS in the low-score group but not significant (*P* = 0.6, Fig. 4c). In addition, a multivariable Cox regression analysis showed that higher prediction score was significantly associated with a higher risk of death (hazard ratio (HR) = 1.087, 95% confidence interval (CI) = 1.01–1.161, *p* = 0.01), independent of known demographic and clinical prognostic factors of PDA including age at surgery, tumor stage, and molecular subtypes of PDA. For the ICGC cohort, there was a better OS in the low-score group than in the high-score group (log-rank test *P* = 0.06; median OS = 25.6 in the low-score group and 13.7 in the high-score group; Fig. 4d), and the raw prediction score was again significantly associated with OS (HR = 1.026, 95%CI = 1.001–1.051), independent of age and tumor stage. Although a further validation analysis of patient’s drug treatment data is needed, our observations suggested that the PDXGEM predictor is predictive of response to gemcitabine, but may have a prognostic value in terms of predicting long-term outcome OS in patients with PDAC.

### PDXGEM for predicting response to 5FU in colorectal cancer patients

5-fluorouracil (5FU) is widely used to treat solid tumors, including colorectal, breast, and head and neck cancer. Using PDXGEM, we built a gene signature to predict response to 5FU among patients with colorectal cancer (CRC) by analyzing data of 16 colorectal cancer PDXs on gene expression and percent of change in tumor volumes after treatment with 5FU. At the drug sensitivity biomarker discovery step, expression levels of 848 probesets were significantly correlated with the percent of change in tumor volumes (nominal *P* < 0.05). We next identified 332 CCE biomarkers from the CCEA of the PDXs and a cohort of metastatic CRC (mCRC) patients (GSE14095 [34]; *n* = 189). In the following RF prediction training step, all the CCE biomarkers displayed positive variable importance and a resultant RF predictor yielded an almost perfect correlation coefficient of 0.978 between PDXGEM scores and observed tumor volume changes in all the 16 PDX models. According to a gene ontology analysis of the biomarkers, the most significantly enriched function was amino acid catabolic process, which is in agreement with that 5-FU drug pathway is regulated via a complex network of anabolic and catabolic genes [35] (Supplementary Table 1).

**Fig. 5:**
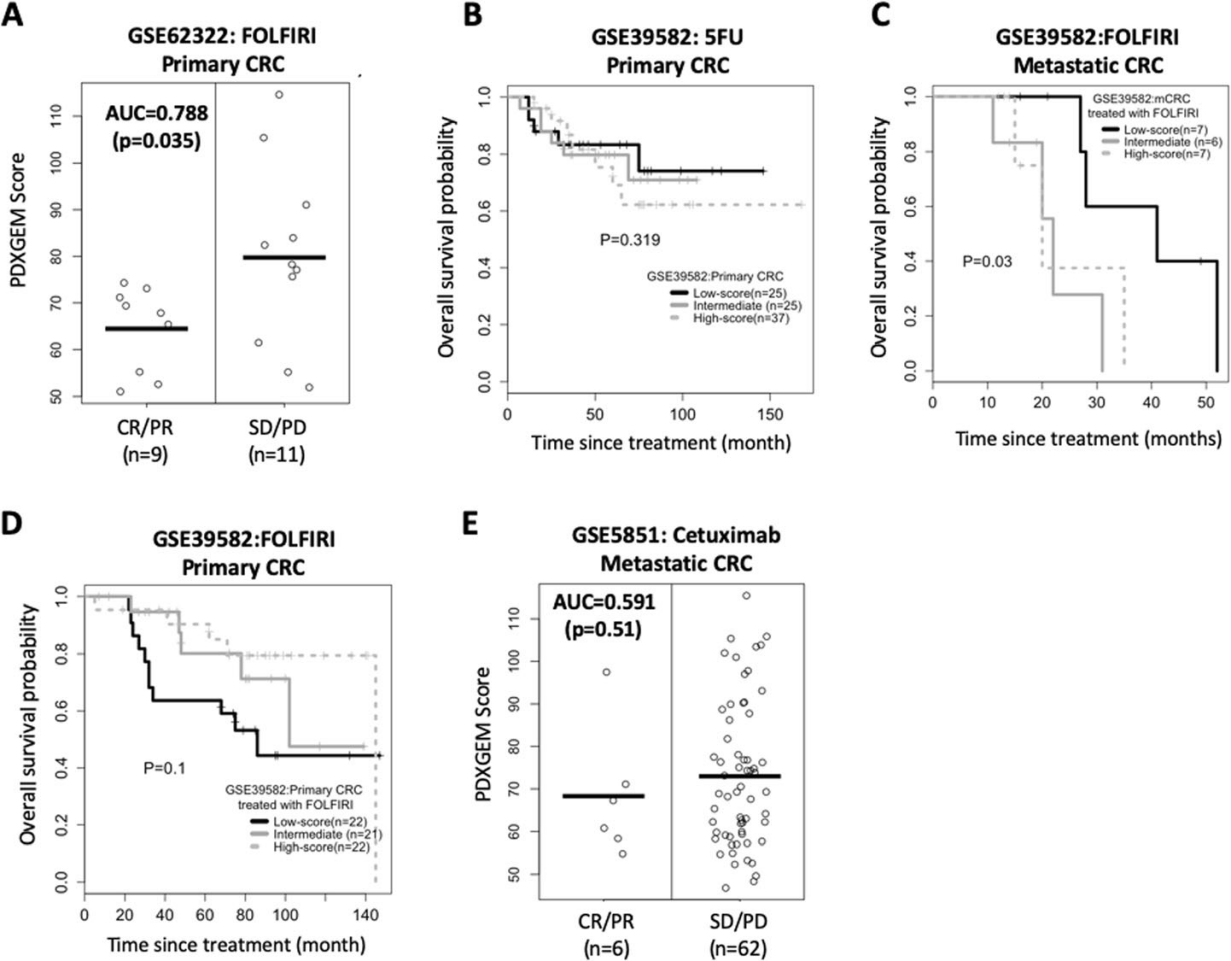
PDXGEM for 5FU response prediction in colorectal cancer patients. **a** Distribution of PDXGEM scores (Y-axis) between responsive and non-responsive patients after at a treatment with 5FU-based chemotherapy. **b**-**d** Kaplan-Meier curves of overall survival for the high (dotted gray), intermediate (gray), and low (black) score group in (**b**) primary colorectal cancer (CRC) patients receiving 5-FU monotherapy in GSE39581 (**c**), and metastatic CRC patients receiving FOLFIRI monotherapy in GSE39581 (**d**) and primary CRC patients receiving FOLFIRI in GSE39581. Prediction scores were broken down at their tertiles. The *P* value was calculated using a survival trend test. **e** Distribution of PDXGEM scores (y-axis) of CRC patients who did not received 5-FU. The P value was calculated using Tarone’s trend test

As an external validation for the prediction performance of the RF predictor, we tested the RF predictor by using two gene expression datasets of CRC patients. The first dataset (GSE62322 [36]) was obtained from a phase 2 clinical trial, in which a percent of change in lesion size was assessed among 20 patients with liver metastatic CRC after receiving FOLFIRI (leucovorin calcium, 5FU, and irinotecan). Our RF predictor produced prediction scores with a significantly large difference between 9 responders and 11 non-responders (Fig. 5a; AUC = 0.788, 95% CI = 0.56–0.99, *P* = 0.035). The other validation dataset was collected from a retrospective study (GSE39582 [37]) and consisted of two CRC patient cohorts: 1) 75 primary CRC patients treated with 5FU monotherapy, and 2) 69 primary and 20 mCRC patients who received 5FU as either FOLFIRI or FOLFOX (leucovorin calcium, 5FU, and oxaliplatin) combination therapies [37]. We divided patients into three balanced groups (low-, intermediate-, and high-score groups) by separating their PDXGEM scores into tertiles and examined survival trends across the three groups. In the 5FU monotherapy cohort, there was a trend of longer OS in primary CRC patients with lower PDXGEM scores; however, this trend was not statistically significant, which might be due to a low event rates (trend test *P* = 0.319) (Fig. 5b). In the combination therapy cohort, we observed a significant trend of a lower score toward an enhanced survival (Tarone’s trend test P = 0.03; median OS = 41, 22, and 20 months for high-, intermediate-, and low-score strata, respectively; see Fig. 5c). In a pairwise comparison of survival between the three groups, we observed a significant difference between the low-score group and the intermediate-score groups (log-rank test *P* = 0.033) and a borderline significant difference between the low-score group and the high-score group (*P* = 0.063). No significant difference was observed between the intermediate- and high-score groups (*P* = 0.56). However, a completely reversed survival trend was observed among the 69 patients with primary, reflecting a known fact that adjuvant FOLFIRI is ineffective in treating resected primary cancer but effective in treating metastatic disease [38, 39] (Fig. 5d).

Finally, we examined the prediction performance of the RF predictor for 5-FU specific response using data obtained from a cohort of mCRC patients in a prospective clinical trial of cetuximab monotherapy (GSE5851 [40]). No significant difference in prediction scores was found (AUC = 0.59; *P* = 0.51), which shows that the PDXGEM predictor is predictive of 5FU-specific response (Fig. 5e).

### PDXGEM for predicting Cetuximab response in colorectal cancer patients

Cetuximab is a monoclonal antibody that targets the epidermal growth factor receptor (EGFR). It was approved for treating patients with EGFR-expressing mCRC without KRAS mutations. Given that around 40% of patients with KRAS wild-type tumors are unresponsive to this targeted therapy, there is an unmet need to identify additional relevant predictive biomarkers beyond KRAS mutations status [41]. To address this need, we used the PDXGEM to construct a predictive multi-gene signature of cetuximab response in patients with mCRC.

We selected 997 differentially expressed probesets via unpaired *t*-test analyses of nine sensitive and seven resistant PDXs after receiving cetuximab therapy (nominal *P* < 0.05). We then screened 670 biomarkers that were concordantly co-expressed across the PDXs and a cohort of mCRC patients (GSE14095 [34]). We constructed an optimal RF predictor based on 585 CCE biomarkers and observed a strong correlation coefficient of 0.98 (*P* < 0.01, *n* = 16) between prediction scores and observed percent of change in tumor volumes in the PDX training dataset.

**Fig. 6:**
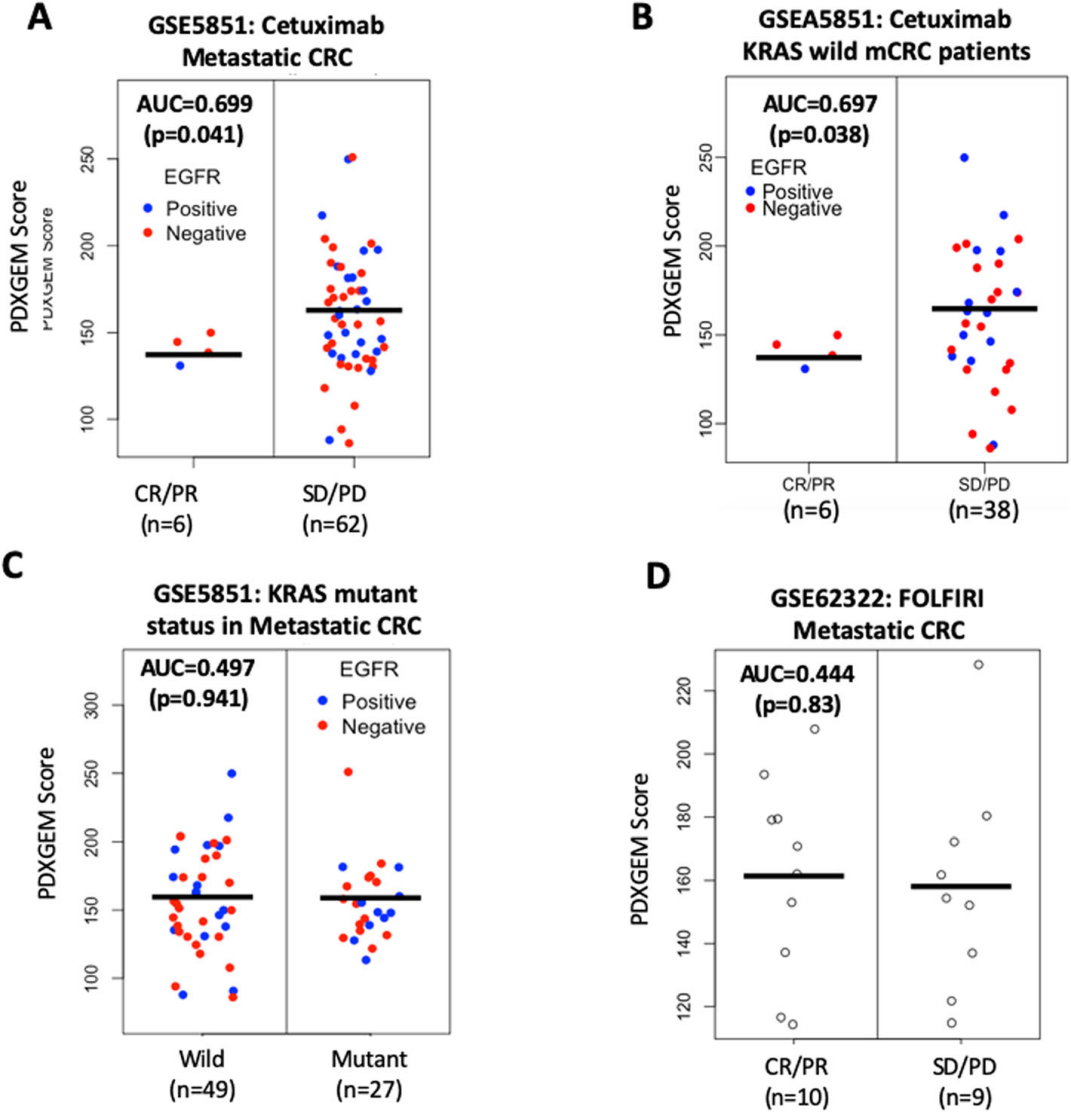
PDXGEM prediction for response to Cetuximab in metastatic colorectal cancer patient. **a** Distribution of PDXGEM scores (y-axis) is compared between metastatic colorectal cancer patients with complete response (CR) or partial response (PR) and those with stable of disease (SD) or progressive disease (PD) after treatment with cetuximab. Blue and red dots are subjects with or without positive epidermal growth factor receptor (EGFR) expression, respectively. **b** PDXGEM scores stratified by KRAS mutation status. **c** Kaplan-Meier curves of overall survival in metastatic colorectal cancer patients who did not receive cetuximab

We proceeded to conduct an external validation study using data from 68 mCRC patients who received cetuximab monotherapy in a phase 2 clinical trial (GSE5851 [40]). We observed a significant difference in prediction scores between 6 responders and 62 non-responders (AUC = 0.699, *P* = 0.041; Fig. 6a). When patients’ survival outcomes were analyzed as described in the prior 5FU PDXGEM study, the high-score group showed worse progression-free survival with 6-months PFS rate of 3.7%, compared with the low- and intermediate-score groups, which had 6-months PFS rates of 18.5 and 19.2%, respectively (Supplementary Fig. 6A; log-rank *P* = 0.085). Moreover, in a subgroup analysis restricted to patients with wild-type KRAS, a significant difference in PDXGEM score was observed between responders and non-responders (*p* = 0.038; Fig. 6b).

Because EGFR-expressing mCRC patients with wild-type KRAS is a part of the drug indication of cetuximab, we examined whether the PDXGEM score was associated with either EGFR expression level or the mutation status of the KRAS gene in the GSE5851 cohort. There was no significant correlation between the PDXGEM score and EGFR expression level (*r* = − 0.103, *P* = 0.41, Supplementary Fig. 6b). No significant difference was observed in PDXGEM scores between patients with wild-type KRAS and those with mutant KRAS (*P* = 0.941, Fig. 6c).

To determine whether the predictor has cetuximab specificity, we validated it using data from an independent cohort of mCRC patients (GSE62322 [36]) who received FOLFIRI but not cetuximab. No significant difference was seen in PDXGEM scores between 9 responders and 10 non-responders (AUC = 0.444; *P* = 0.72; Fig. 6d), suggesting that the predictor is specifically predictive of response to cetuximab.

### PDXGEM signature predictive of Erlotinib response in NSCLC patients and cell clines

Erlotinib is an EGFR tyrosine kinase inhibitor that was approved for the treatment of non-small cell lung cancer (NSCLC), but its overall therapeutic efficacy is minimal [42]. We constructed a multi-gene expression signature to predicting response to the erlotinib by analyzing data on the pretreatment gene expression profiles and percent of changes in tumor volume in 8 NSCLC PDXs following erlotinib administration.

We screened 1624 initial drug sensitivity biomarkers were screened using an unpaired *t*-test that compared three PDXs with tumor shrinkage to five PDXs with tumor growth. Among them, 112 biomarkers showed concordant co-expression patterns between the PDXs and a cohort of 150 NSCLC patients (GSE43580 [43]). Finally, a 106-gene based RF predictor predictive of post-erlotinib treatment tumor volume change was trained with all the PDXs. PDXGEM score from the RF predictor was significantly correlated with the observed percent of change in tumor volume in the PDX training set (*r* = 0.973 and *P* < 0.01; *n* = 8).

**Fig. 7:**
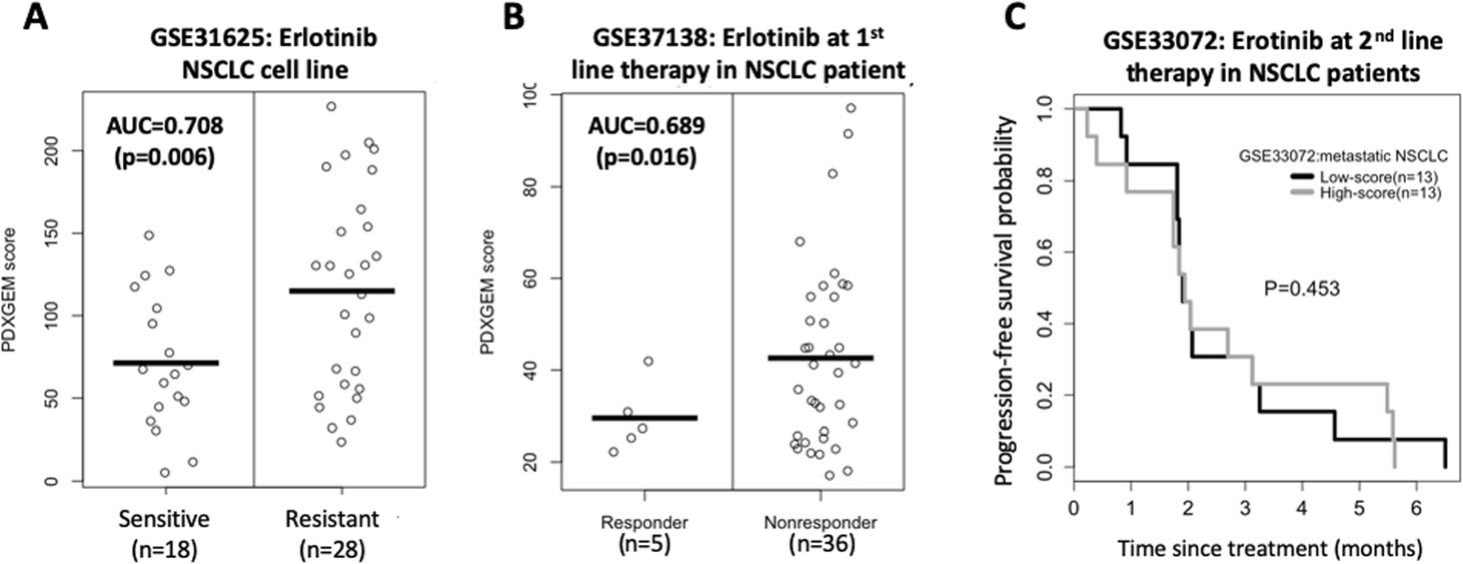
PDXGEM prediction for response to erlotinib in non-small cell lung cancer (NSCLC) patient. PDXGEM scores (**a**) between erlotinib-sensitive and erlotinib-resistant NSCLC cell lines, and (**b**) between the NSCLC patients who were responsive and those who were nonresponsive to erlotinib in the first line setting (**c**) Progression-free survival curves in metastatic NSCLC patients who receive erlotinib as the second-line treatment setting

To validate the prediction performance of the RF predictor, we generated PDXGEM scores for in vitro erlotinib-treated NSCLC cell lines (GSE31625 [44]; *n* = 46). There was a significantly large difference in PDXGEM scores between 18 erlotinib-sensitive cell lines and 28 erlotinib-resistant cell lines (AUC = 0.708 and *P* = 0.006; see Fig. 7a). We next validated the RF predictor on data from a prospective clinical trial cohort of 41 refractory NSCLC patients who received the first-line treatment with erlotinib in combination with bevacizumab (GSE37138 [45]). We observed a significant difference in PDXGEM scores between 5 responders and 36 non-responders (AUC = 0.689 and *P* = 0.016; Fig. 7b). To examine whether the RF predictor is also predictive of treatment response at recurrent disease settings, we further validated the predictor on data from 26 patients with relapsed or metastatic NSCLC who had EGFR mutation and received erlotinib as second-line treatment (GSE33072 [46]). Although our predictor yielded the highest prediction scores for 2 patients with the shortest PFS, there was no significant difference in PFS between the high-score group and the low-score group (Fig. 7c and Supplementary Fig. 7A), indicating that our predictor may not be predictive at the second line treatment setting.

To determine whether the PDXGEM predictor has erlotinib-specificity, we produced PDXGEM scores for 20 patients with the NSCLC subtype lung squamous carcinoma who did not receive erlotinib or other EGFR inhibitors (GSE68793). There was no significant association between prediction scores and PFS or OS (Supplementary Fig. 7B and 7C), showing that the predictor is specifically predictive of response to erlotinb. However, additional studies are needed to further confirm its erlotinib-specificity in patients with other subtypes of NSCLC.

Collectively, our validation results showed that our PDXGEM predictor was predictive of response to erlotinib in refractory NSCLC patients in the first line treatment setting, but not in the second line treatment setting.

## Discussion

Predictive cancer biomarkers are necessary toward a personalized cancer therapy, by which a cancer patient will likely to be treated with the most effective anti-cancer drugs available.

In this study, we developed a statistical bioinformatics pipeline, PDXGEM, to build a multi-gene expression signature as a quantitative cancer biomarker for predicting cancer patients’ responses to a single anti-cancer drug on the basis of data on pretreatment gene expression profiles and posttreatment outcomes in preclinical PDX models. We demonstrated that PDXGEM can build a predictive gene expression signature for cancer patients’ responses to chemotherapy and targeted therapy agents.

Because the PDX tumors can alter the biological characteristics of their origin patient tumors to adapt to new growth environments, we devised CCEC statistics to quantify the degree of concordance of co-expression patterns between preclinical PDX tumors and cancer patient tumors. Although drug sensitivity biomarkers obtained directly from a correlative or differential expression analysis of data from preclinical PDX models could serve as predictive biomarkers by themselves, we showed that a subset of them with significant CCEC was able to induce a more translatable predictor, thereby yielding a better performance of predicting therapeutic outcomes in cancer patients as shown in our examples.

Notably, the PDXGEM does not use any patients’ outcome data during the development of a prediction model whereas various strategies for developing predictive gene signatures by analyzing data of preclinical models often uses patients’ outcome data for screening biomarkers or training a prediction model. The PDXGEM only uses pretreatment gene expression data of cancer patients at the CCEA step. Therefore, the PDXGEM can build gene signatures for even unapproved anti-cancer drugs for patients with a certain type of cancer. Although CCEA was introduced to improve GEMs that are trained by only using PDX model data, PDXGEM still can build a gene expression signatures by skipping the CCEA step in the absence of available pretreatment patient data.

The results of our PDXGEM application showed that significantly predictive GEMs can be developed from a small cohort of PDX models. For example, the PDXGEM for erlotinib only uses 8 PDX models but validation analyses of this model on NSCLC cancer cell lines and on patients with this disease yielded statistically significant prediction performance. This level of predictive performance is a highly desirable and encouraing feature when there is a limited number of available preclinical PDX models.

Indication of targeted therapy agents highly depends on the status of their known target companion biomarkers in patient tumors. Our PDXGEM predictor for cetuximab is able to differentiate the responsive from the nonresponsive even in mCRC patients with wild-type KRAS genes, demonstrating that Integrative usage of PDXGEM along with known companion biomarkers of a targeted therapy has a potential for improving clinical outcomes and thereby the quality of life of a targeted cancer patient population. Moreover, PDXGEM has the potential of being used to develop a predictor of response to a recent breakthrough immunotherapy. Several immune-oncology studies have begun to create and investigate PDX mouse models with human immune system [47]. Data collected from these humanized mice will enable our PDXGEM pipeline to develop predictive cancer biomarkers of response to the immunotherapy.

Many cancer drugs, including those used in our study, are multi-indication drugs that can be used for treating more than one cancer type. For instance, paclitaxel is currently a standard chemotherapy drug for treating breast cancer and ovarian cancer. There is great interest in identifying a new treatment indication of existing anti-cancer therapy agents. We have recently introduced a drug repositioning approach (CONCORD) to translating predictive cancer biomarkers from one cancer type to another [13]. The CONCORD framework was used to analyze the gene expression and drug sensitivity data of a large panel of cancer cell lines with different types of cancer. Similarly, given that more PDX panels that span multiple types of cancer are becoming publicly available, there will be great interest in using PDXGEM to explore a drugs’ potential for anti-cancer drug repositioning by testing prediction values of a predictive gene expression signature across multiple types of cancer.

There are clear challenges and opportunities in developing the PDXGEM pipeline. A PDXGEM predictor for only one drug may just provide limited information on whether a patient will be likely to be cured with the drug or not. However, if PDXGEM predictors are built for a multitude of FDA-approved anticancer drugs and then be used simultaneously for evaluating comparative effectiveness among drugs, it may enable to choose most beneficial drug to treat a cancer patient in advance.

In the drug sensitivity gene discovery step, we performed *t*-test to identify differentially expressed genes between PDXs with shrunken tumor volumes and PDXs with increased tumor volumes because we presumed that these genes would bear biologically reliable information regarding the pharmacogenomic mechanism that inhibits tumor growth and kills tumor cells. When a group sample size was less than three, we used a correlation analysis instead of t-test, as this test can lose a statistical power in microarray data analysis due to small sample sizes. However, this approach may not be optimal. More sophisticated bioinformatics methods such as limma [48] or recent popular deep-learning algorithms that do not involve any feature selection step may provide a better set of candidate predictive genes. Evaluating their performances in developing an optimal biomarker discovery method or guideline would constitute exciting future research topics.

The results of our concordant co-expression analysis were dependent on a pretreatment gene expression data set of cancer patients that represented a cancer type of interest. Although we used the largest gene expression dataset available, in terms of the number of patients and the coverage of histological cancer subtypes, merging multiple independent gene expression datasets would allow for a more comprehensive gene expression dataset of individual cancer subtypes. We built a multi-gene expression model using the RF modeling algorithm to handle a larger number of gene biomarkers than the smaller sample size of the PDX data as a model training data. However, other statistical prediction modeling and machine learning algorithms such as penalized linear regression and support vector machine analyses could also be used to build more accurately predictive models [49]. The majority of gene expression datasets we analyzed was profiled on microarray platforms. We validated the PDXGEM signature for gemcitabine on gene expression data that were profiled using RNA sequencing (RNAseq) platform (ICGC cohort). However, validating the signature’s cross-platform prediction performance on other next generation sequencing datasets is warranted. Furthermore, a recent pharmacogenomics study of cancer cell lines reported that transcript-level expression data profiled on the RNAseq platform could lead to more predictive biomarkers than gene-level expression data. The application of PDXGEM to RNAseq transcriptional profiling data may also lead to a better performing predictive cancer biomarker.

In developing a predictive biomarker, it is important to evaluate whether the biomarker is specifically predictive of a drug of interest. We thus validated the final PDXGEM of a drug on patients who were not treated with the drug. However, we were unable to perform the validation study on multiple datasets due to a lack of available data; the majority of drugs analyzed in our study are core components of current standard of care regimens. Furthermore, although many cancer treatment regimens are combinations of multiple chemotherapy drugs, but our current PDXGEM study is limited to the prediction of response to a single drug. Further research is warranted to develop a drug predictor of response to combination chemotherapy based on data obtained from PDXs treated with a single drug.

It will be useful to investigate whether PDXGEM can be extended to different molecular platform data such as genome-wide genetic variant data, proteomics data, and metabolomics data. The mathematical framework of PDXGEM will be broadly applicable to these different molecular platforms. However, one may need to carefully examine whether large, reliable patient data resources are available and whether predictive therapeutic biomarkers can be obtained from such molecular profile data. Another promising research focus is to predict in advance a post-treatment adverse events (AEs) on the basis of gene expression data; in cancer treatment, an AE is also an important post-treatment outcome along with response and survival. A part of our PDXGEM pipeline, such as an initial biomarker (feature) selection and multi-gene expression model training, will be directly applicable to identifying AE-correlated genes and training a multi-gene expression model.

Lastly, we developed a web-based PDXGEM application (http://pdxgem.moffitt.org) to share the PDXGEM algorithm with the scientific community, in the hope that this tool will allow researchers to gain a better understanding of the drug targets and validation in a prospective study.

## Conclusions

Molecular gene expression profiles and drug activity data from PDX tumors can be used to develop highly predictive cancer biomarkers for predicting responses to anti-cancer drugs in cancer patients. The clinical utility of PDXGEM predictions should be assessed in a prospective study.

## Materials and methods

### Gene expression data and anti-cancer response data of PDX and cancer patient cohorts

Data on gene expression profiles and post-treatment percent change in tumor volume of the Novartis PDX panel were obtained from Gene Expression Omnibus (GEO) repository (https://www.ncbi.nlm.nih.gov/geo/). The gene expression data and clinical outcome data of cancer patients used for the CCEA or validation analyses were also publicly available at GEO (http://www.ncbi.nlm.nih.gov/geo) as well as ArrayExpress (https://www.ebi.ac.uk/arrayexpress/) and ICGC (https://dcc.icgc.org/repository). A descriptive summary and accession ID of all the data can be found in Supplementary Table 2**.**

### In vivo PDX-based drug sensitivity biomarker discovery

We discovered genes whose expression levels were significantly associated with in vivo activities of each anti-cancer drug administered to the PDX tumors of the target cancer type. Drug activity was calculated as a percent change in PDX tumor volumes (= 100 x (post-treatment tumor volume – pretreatment tumor volume) / pretreatment tumor volume). A negative drug activity value for a PDX, thus, indicates a tumor shrinkage, and a positive drug activity value represents tumor growth. Drug activity data and pretreatment gene expression profiling data of the PDX models were analyzed to screen initial drug sensitivity biomarkers. The basic unit of the biomarkers was an individual probeset on the PDX microarray. Drug sensitivity biomarkers were selected using an unpaired two sample *t*-test that quantifies differential gene expression levels between PDXs with shrunken tumors and those with grown tumors. When a sample size in one of the two PDX groups was less than three and a variation in tumor volume changes was near zero, we used a correlation analysis of gene expression levels with the percent change in tumor volumes to screen the initial drug sensitivity biomarkers. For both the *t*-test and correlation analyses, all statistical tests were two-sided and the FDR was controlled to be less than 0.05 to correct for multiple comparisons. When no significant genes were found, mainly due to a small sample size of available PDXs, we controlled a less conservative nominal type I error rate of 0.05 to identify initial drug sensitivity biomarkers.

### Concordant co-expression analysis (CCEA)

Because PDX tumors can alter the biological characteristics of their origin patient tumors to adapt to new growth environments, potentially not all the drug sensitivity genes screened in an analysis of PDX tumor data will be predictive of response of cancer patients. To explicitly consider such biological differences, we selected genes with concordant co-expression patterns between the PDX tumors and cancer patient tumors. To quantify the degree of concordance of each gene’s co-expression relationships, we calculated the concordance co-expression coefficient (CCEC) for each gene as follows: using gene expression data from each of the two cancer systems separately, we first constructed two *n × n* correlation matrices for *n* initial drug-sensitivity biomarkers; we denoted the two correlation matrices, e.g. one for the PDX tumor set and the other for the pretreatment cancer patient tumor set, as *U = [U*_*ij*_*]*_*n × n*_ and *V = [V*_*ij*_*]*_*n × n*_, where *U*_*ij*_ and *V*_*ij*_ were the correlation coefficients between gene *i* and *j* in the PDX set and the patient tumor set, respectively; and the CCEC for the gene *g*, *c(g),* is derived as

$$ c(g)=\frac{2{\sum}_{k\ne g}\left({U}_{kg}-\overline{U_{.g}}\right)\left({V}_{kg}-\overline{V_{.g}}\right)}{\sum_{k\ne g}{\left({U}_{kg}-\overline{U_{.g}}\right)}^2+{\sum}_{k\ne g}{\left({V}_{kg}-\overline{V_{.g}}\right)}^2+{\sum}_{k\ne g}{\left(\overline{U_{.g}}-\overline{V_{.g}}\right)}^2} $$

where $$ \overline{{\mathrm{U}}_{.\mathrm{g}}}=\frac{1}{\mathrm{n}-1}{\sum}_{\mathrm{k}\ne \mathrm{g}}{\mathrm{U}}_{\mathrm{k}\mathrm{g}} $$ and $$ \overline{{\mathrm{V}}_{.\mathrm{g}}}=\frac{1}{\mathrm{n}-1}{\sum}_{\mathrm{k}\ne \mathrm{g}}{\mathrm{V}}_{\mathrm{k}\mathrm{g}} $$.

Briefly, the CCEC *c(g)* first computed two vectors of gene-gene correlation coefficients. One vector consisted of correlation coefficients of gene *g* with other *n-1* genes for the PDX tumor set. The other vector was computed in the same manner for the patient tumor set. The CCEC next quantifies the degree of agreement between the two vectors by calculating Lin’s concordance correlation coefficient [50]. Therefore, in the example of paclitaxel, *c(g)* reflects the degree of concordance between the breast cancer PDX panel and GSE3494 breast cancer patient cohort for expression relationships of probeset *g* with other *n-1* probesets. If *c(g)* took a statistically significant positive value under an FDR of 0.05, then probeset *g* was selected as a CCE biomarker. Because the probeset *g* was initially selected among *n* drug-sensitivity biomarkers, the probeset still retained a significant association with drug sensitivity. To compute CCEC, we used ‘epi.ccc’ function that was implemented in epiR package in R program. The *P*-value for the concordance correlation coefficient was corrected for multiple testing by using the Benjamini-Hochberg method implemented in ‘p.adjust’ function.

### PDXGEM modeling and evaluation

A multi-gene expression model for predicting each drug’s response was built using gene expression data and drug activity data of the PDX panel that was used in the above drug sensitivity biomarker discovery. The drug activity data and gene expression data of the PDX model for all CCE biomarkers, defined as drug sensitivity genes with statistically significant CCEC, formed the model training data. After completing a gene-wise standardization of the model training data, we performed a random forest classification and regression analysis using the ‘randomforest’ function implemented in randomForest package at the default setting in R program. The prediction performance of the resultant RF predictor was first evaluated by calculating a correlation coefficient between the observed and predicted tumor volume changes in the PDX models. When there was a significant correlation relationship, the RF predictor was validated on gene expression data and post-treatment clinical outcome data of cancer patient cohorts that were independent of the biomarker discovery and the prediction model development.

### PDXGEM prediction and validation

To validate the prediction performance of each drug’s final RF prediction model, we produced prediction scores of the RF model (PDXGEM score) for cancer patient cohorts that were not involved in either drug sensitivity biomarker discovery or prediction model development procedures. The performance of each drug’s PDXGEM prediction was then assessed in a prospective manner. For cancer patient cohorts with binary response outcome data, we compared prediction scores between responsive and non-responsive patient groups by performing a two-sample *t*-test. The AUC was also calculated to summarize an overall prediction accuracy of the prediction model. For cancer patient cohorts with survival outcome data, survival distributions were compared between their prediction score strata via Kaplan-Meier analysis, log-rank test, and Tarone’s trend test. Multivariable Cox proportional hazard regression analysis was also used to examine an association between raw continuous prediction scores and survival outcomes. All survival analyses were performed using survival and survMiner packages in R program.

### Gene ontology analysis

To assess any potent functional behaviors and mechanisms by which the multi-gene expression model could predict patients’ responses to an anticancer drug of interest, we selected CCE biomarkers showing a positive value of variable importance in the RF analysis. In brief, the variable importance is a model selection measure by summarizing the difference in prediction accuracy between two RF predictors with and without individual biomarkers. Finally, web-based Enrichr tool was used to conduct GO analysis by submitting a set of CCE biomarkers with positive values of variable importance measure [51].

## Supplementary information

**Additional file 1: Supplementary Figure 1.** File type: PDF. Distribution of pairwise gene-gene correlation coefficients at varying concordant co-expression coefficient (CCEC) values. **Supplementary Figure 2.** File type: PDF. Variable importance of biomarkers in paclitaxel PDXGEM. **Supplementary Figure 3.** File type: PDF. Prediction scores of paclitaxel PDXGEM in breast cancer patients. **Supplementary Figure 4.** File type: PDF. Prediction scores of Paclitaxel PDXGEM built by skipping CCEC analysis. **Supplementary Figure 5.** File type: Prediction scores of trastuzumab PDXGEM in breast cancer patients who were not treated with trastuzumab. **Supplementary Figure 6.** File type: PDF. *PDXGEM for cetuximab in colorectal cancer patients*. **Supplementary Figure 7.** File type: PDF. PDXGEM for erlotinib in non-small cell lung cancer patients

**Additional file 2: Supplementary Table 1.** File type: Excel. Gene ontology and annotation analysis result of PDXGEM biomarkers.

**Additional file 3: Supplementary Table 2.** File type: Word. The list of gene expression and anti-cancer drug response data sets

## Data Availability

The datasets analyzed during the current study are available with data accession IDs presented in the manuscript in the Gene Expression Omnibus repository, https://www.ncbi.nlm.nih.gov, and ArrayExpress repository, https://www.ebi.ac.uk/arrayexpress. The data that support the findings of this study are available at http://pdxgem.moffitt.org/publications. R scripts and functions are also available at http://github.com/ykim5g/PDXGEM.

## Abbreviations

5FU: 5-fluoracil
AE: Adverse Event
AUC: Area under Receiver Operating Characteristic Curve
CCEA: Concordance Co-Expression Analysis
CCEC: Concordance Co-Expression Coefficient
CRC: Colorectal Cancer
FAC: Fluorouracil, doxorubicin, and cyclophosphamide
GEM: Gene Expression Model
GO: Gene Ontology
HER2: Human Epidermal Growth Factor Receptor 2
ICGC: International Cancer Genome Consortium
mCRC: Metastatic Colorectal Cancer
NSCLC: Non-Small Cell Lung Cancer
OS: Overall Survival
pCR: Pathological Complete Response
PD: Progressive Disease
PDA: Pancreatic Ductal Adenocarcinoma
PDX: Patient-Derived Xenograft
PFS: Progression-Free Survival
PR: Partial Response
RD: Residual of Disease
RF: Random Forest
RNAseq: RNA Sequencing
ROC: Receiver Operating Characteristic curve
SD: Stable Disease
