# Supplementary material for: PDXGEM: patient-derived tumor xenograft-based gene expression model for predicting clinical response to anticancer therapy in cancer patients
Source: BMC Bioinformatics. 2020 Jul 6;21:288. doi: 10.1186/s12859-020-03633-z (PMC7336455; doi:10.1186/s12859-020-03633-z)
Supplement: Supplementary file 1 — Additional file 1: Supplementary Figure 1. File type: PDF. Distribution of pairwise gene-gene correlation coefficients at varying concordant co-expression coefficient (CCEC) values. Supplementary Figure 2. File type: PDF. Variable importance of biomarkers in paclitaxel PDXGEM. Supplementary Figure 3. File type: PDF. Prediction scores of paclitaxel PDXGEM in breast cancer patients. Supplementary Figure 4. File type: PDF. Prediction scores of Paclitaxel PDXGEM built by skipping CCEC analysis. Supplementary Figure 5. File type: Prediction scores of trastuzumab PDXGEM in breast cancer patients who were not treated with trastuzumab. Supplementary Figure 6. File type: PDF. PDXGEM for cetuximab in colorectal cancer patients. Supplementary Figure 7. File type: PDF. PDXGEM for erlotinib in non-small cell lung cancer patients [file 12859_2020_3633_MOESM1_ESM.pptx]

## Slide 1
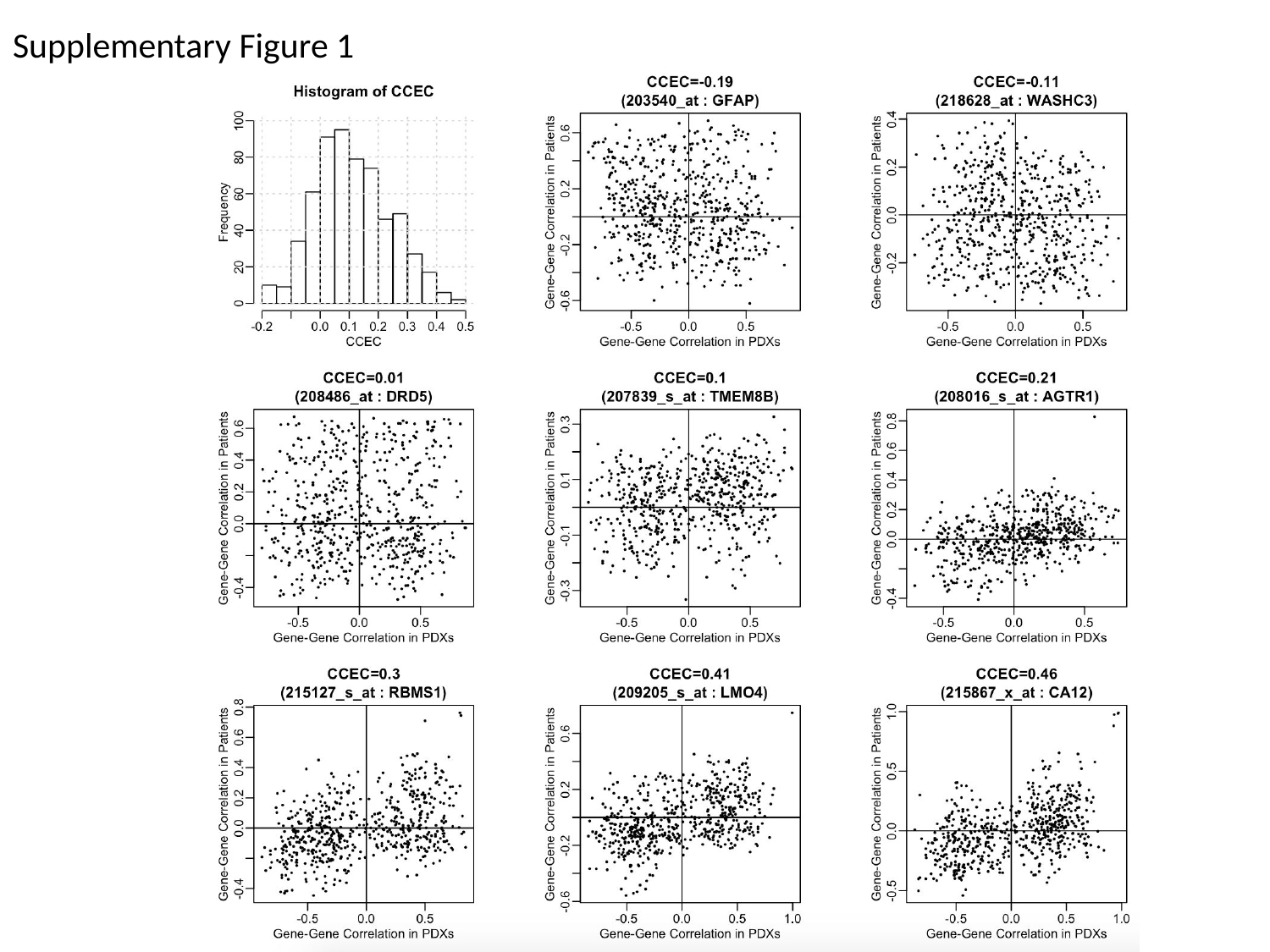

# Supplementary Figure 1

## Slide 2
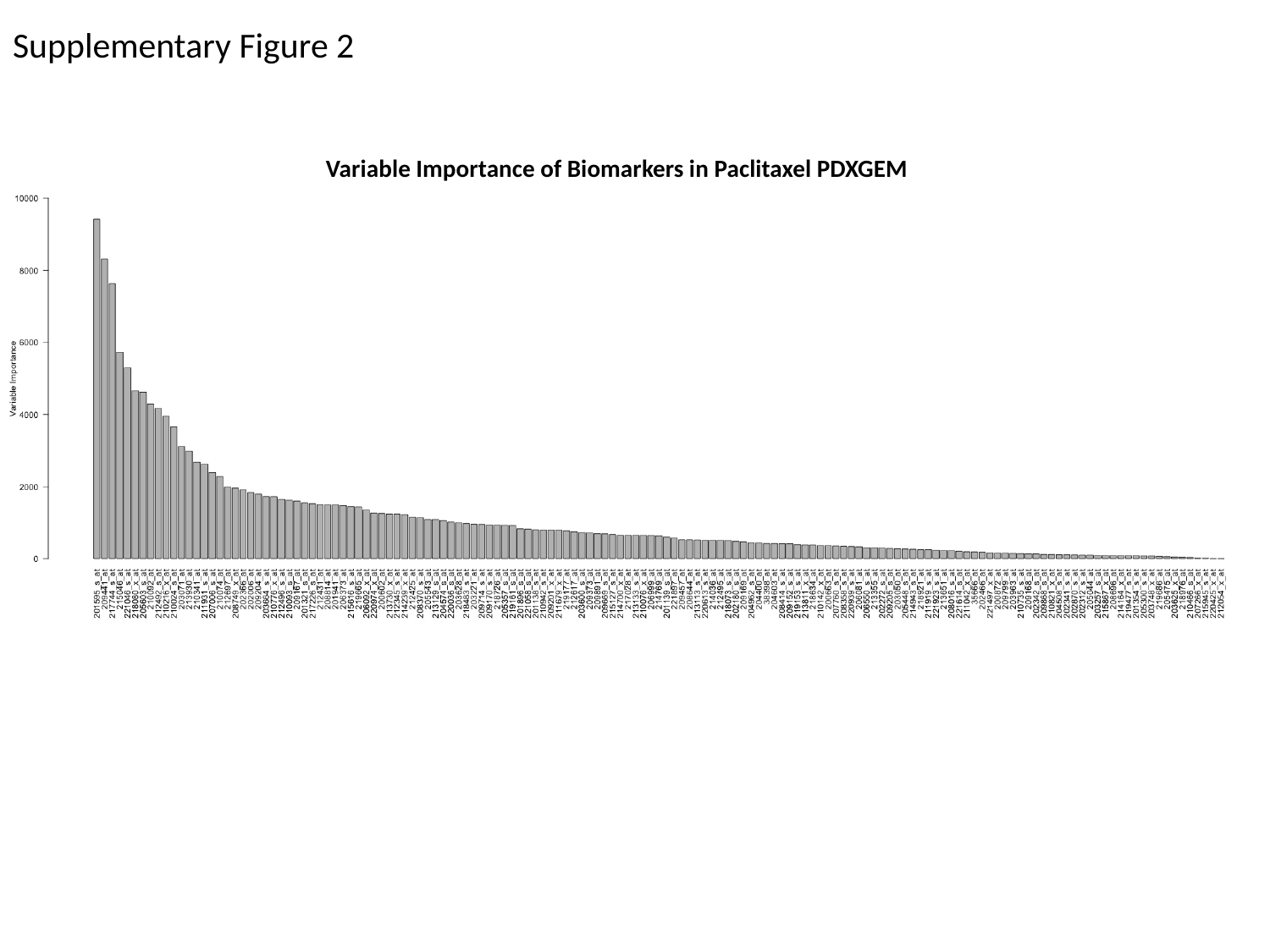

# Supplementary Figure 2
Variable Importance of Biomarkers in Paclitaxel PDXGEM

## Slide 3
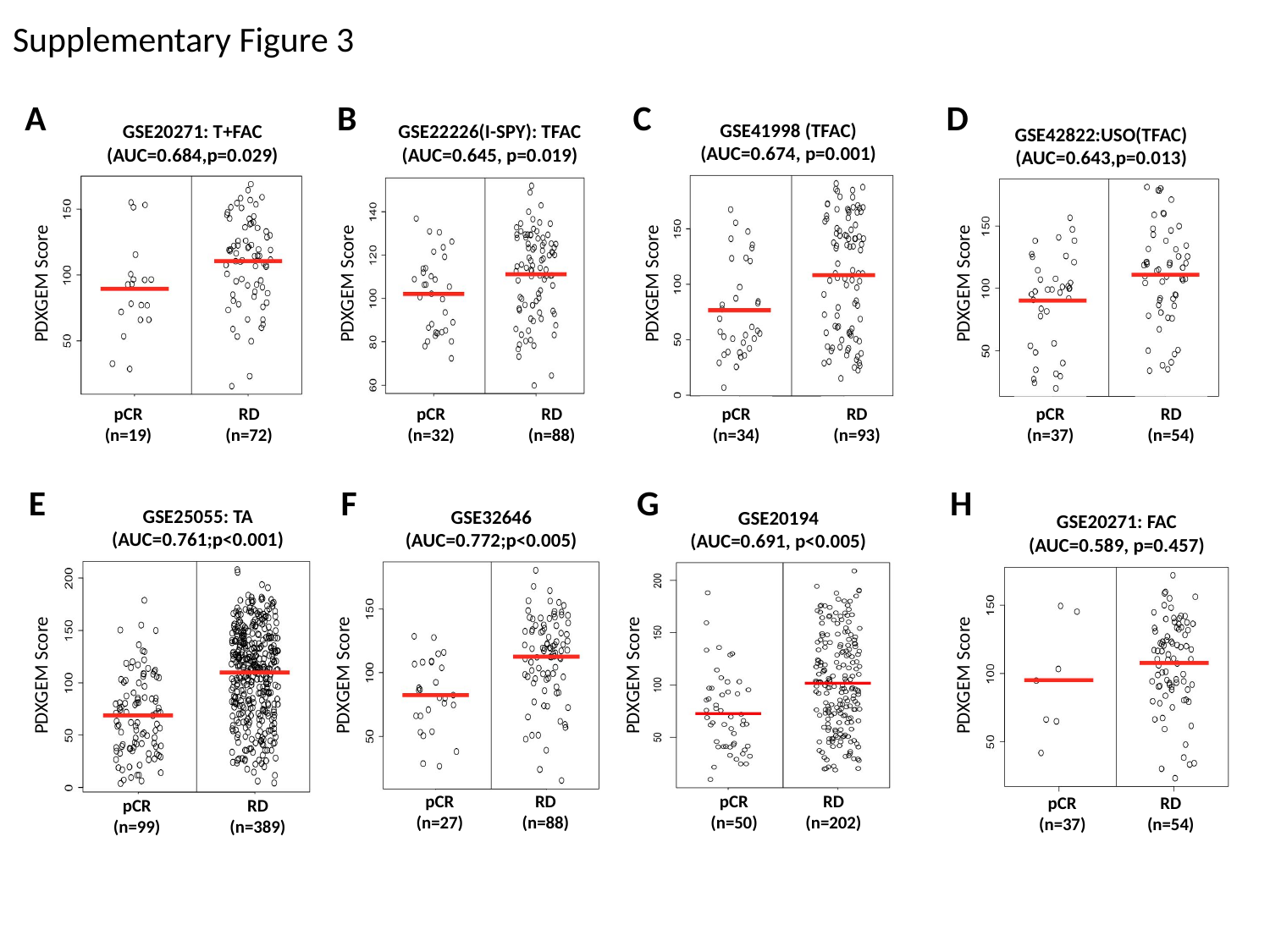

# Supplementary Figure 3
A
B
C
D
GSE41998 (TFAC)
(AUC=0.674, p=0.001)
GSE22226(I-SPY): TFAC
(AUC=0.645, p=0.019)
GSE20271: T+FAC
(AUC=0.684,p=0.029)
GSE42822:USO(TFAC)
(AUC=0.643,p=0.013)
PDXGEM Score
PDXGEM Score
PDXGEM Score
PDXGEM Score
pCR
(n=19)
RD
(n=72)
pCR
(n=32)
RD
(n=88)
pCR
(n=34)
RD
(n=93)
pCR
(n=37)
RD
(n=54)
E
F
G
H
GSE25055: TA
(AUC=0.761;p<0.001)
GSE32646
(AUC=0.772;p<0.005)
GSE20194
(AUC=0.691, p<0.005)
GSE20271: FAC
(AUC=0.589, p=0.457)
PDXGEM Score
PDXGEM Score
PDXGEM Score
PDXGEM Score
pCR
(n=27)
RD
(n=88)
pCR
(n=50)
RD
(n=202)
pCR
(n=37)
RD
(n=54)
pCR
(n=99)
RD
(n=389)

## Slide 4
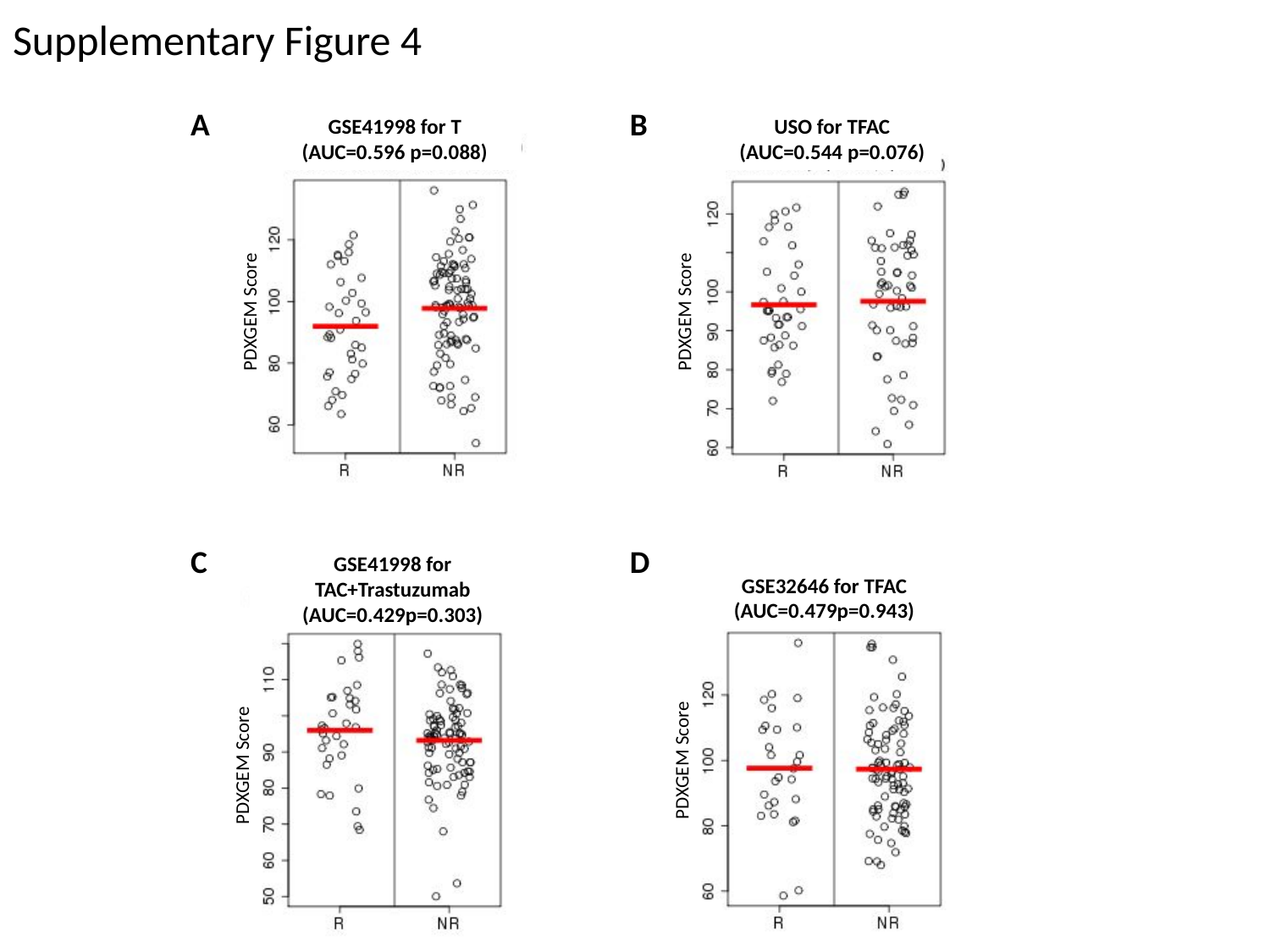

# Supplementary Figure 4
A
B
GSE41998 for T
(AUC=0.596 p=0.088)
USO for TFAC
(AUC=0.544 p=0.076)
PDXGEM Score
PDXGEM Score
C
D
GSE41998 for TAC+Trastuzumab
(AUC=0.429p=0.303)
GSE32646 for TFAC (AUC=0.479p=0.943)
PDXGEM Score
PDXGEM Score

## Slide 5
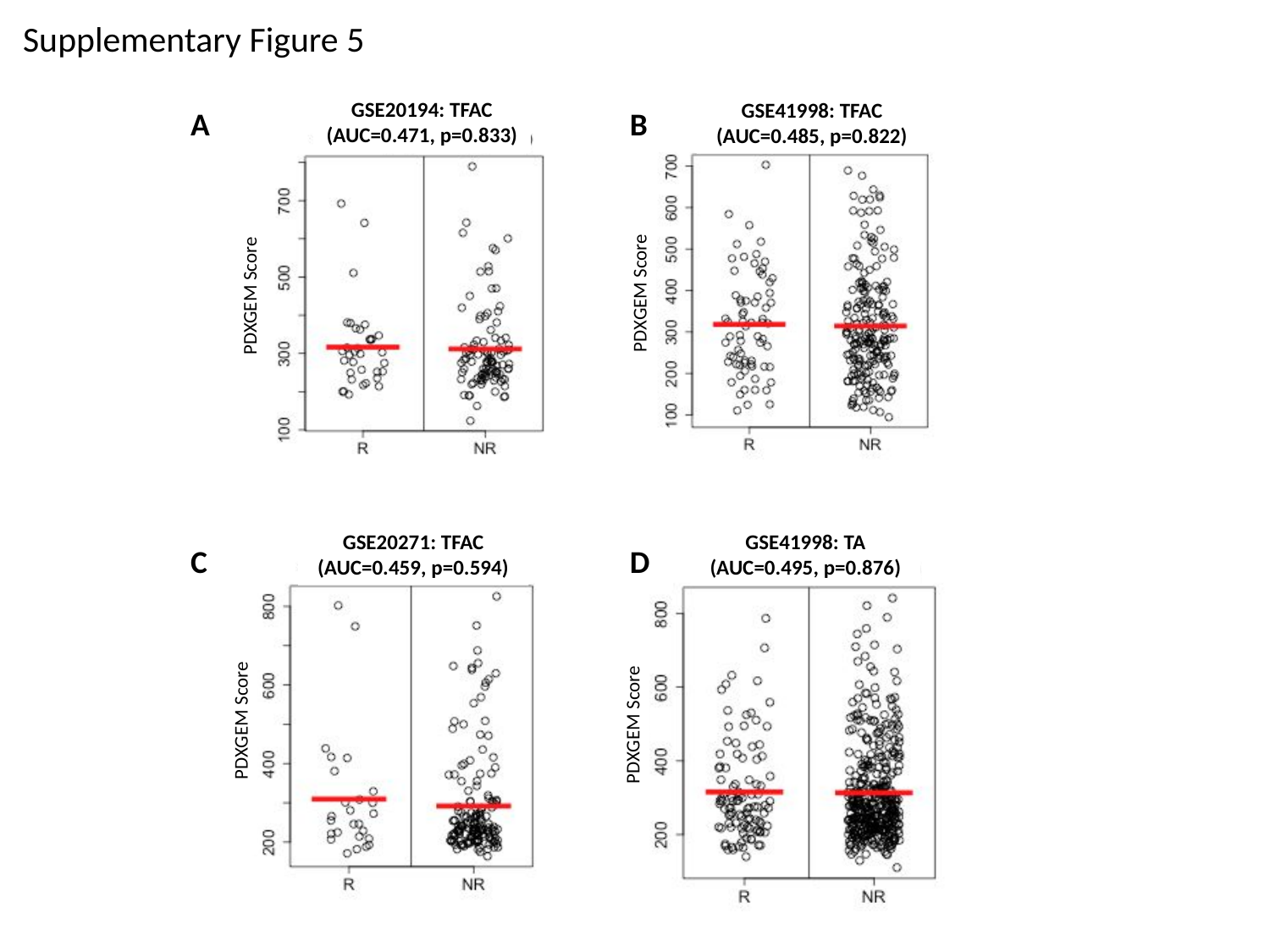

# Supplementary Figure 5
GSE20194: TFAC
(AUC=0.471, p=0.833)
GSE41998: TFAC
(AUC=0.485, p=0.822)
A
B
PDXGEM Score
PDXGEM Score
GSE41998: TA
(AUC=0.495, p=0.876)
GSE20271: TFAC
(AUC=0.459, p=0.594)
C
D
PDXGEM Score
PDXGEM Score

## Slide 6
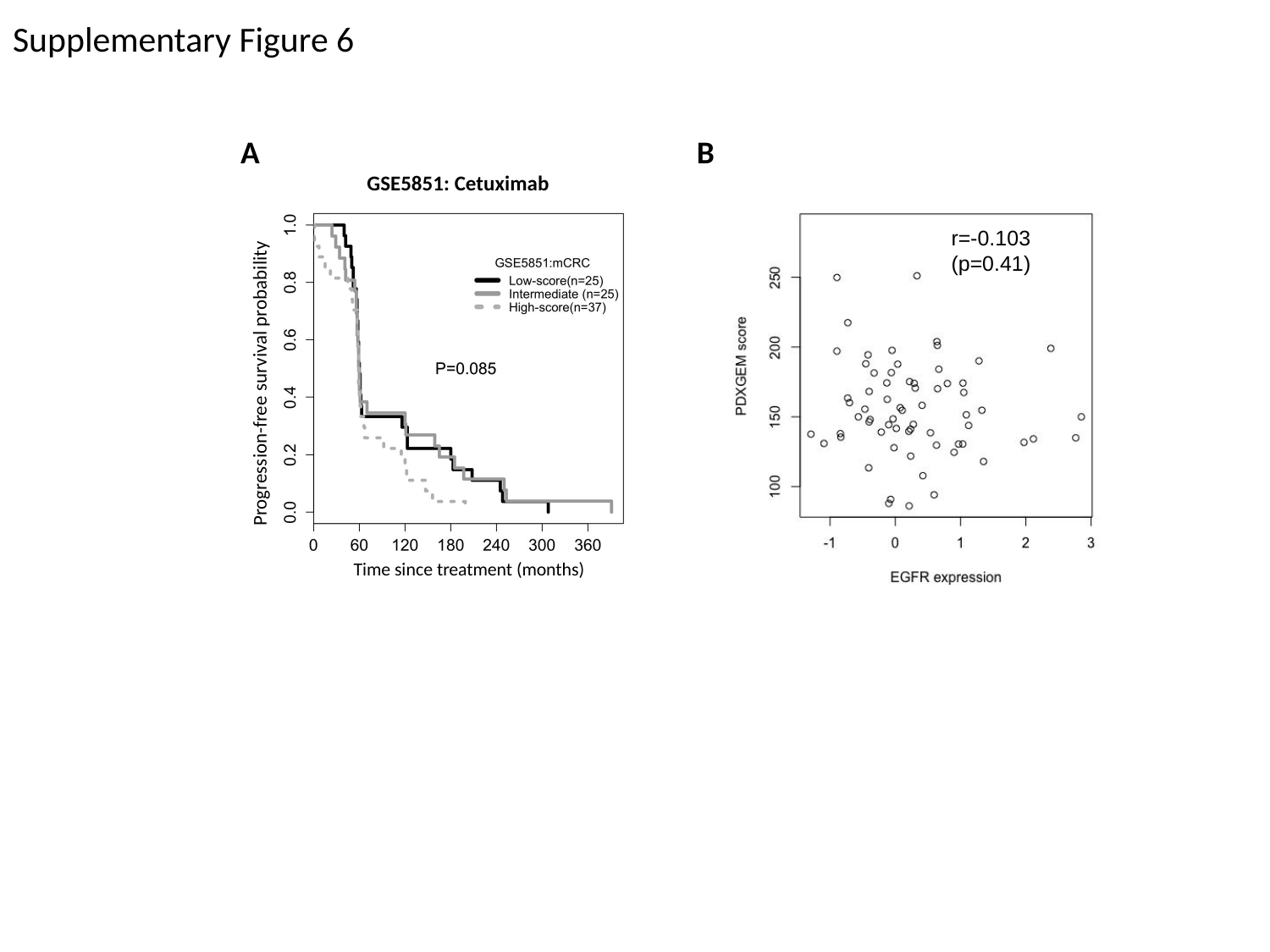

# Supplementary Figure 6
A
B
GSE5851: Cetuximab
r=-0.103
(p=0.41)
Progression-free survival probability
Time since treatment (months)

## Slide 7
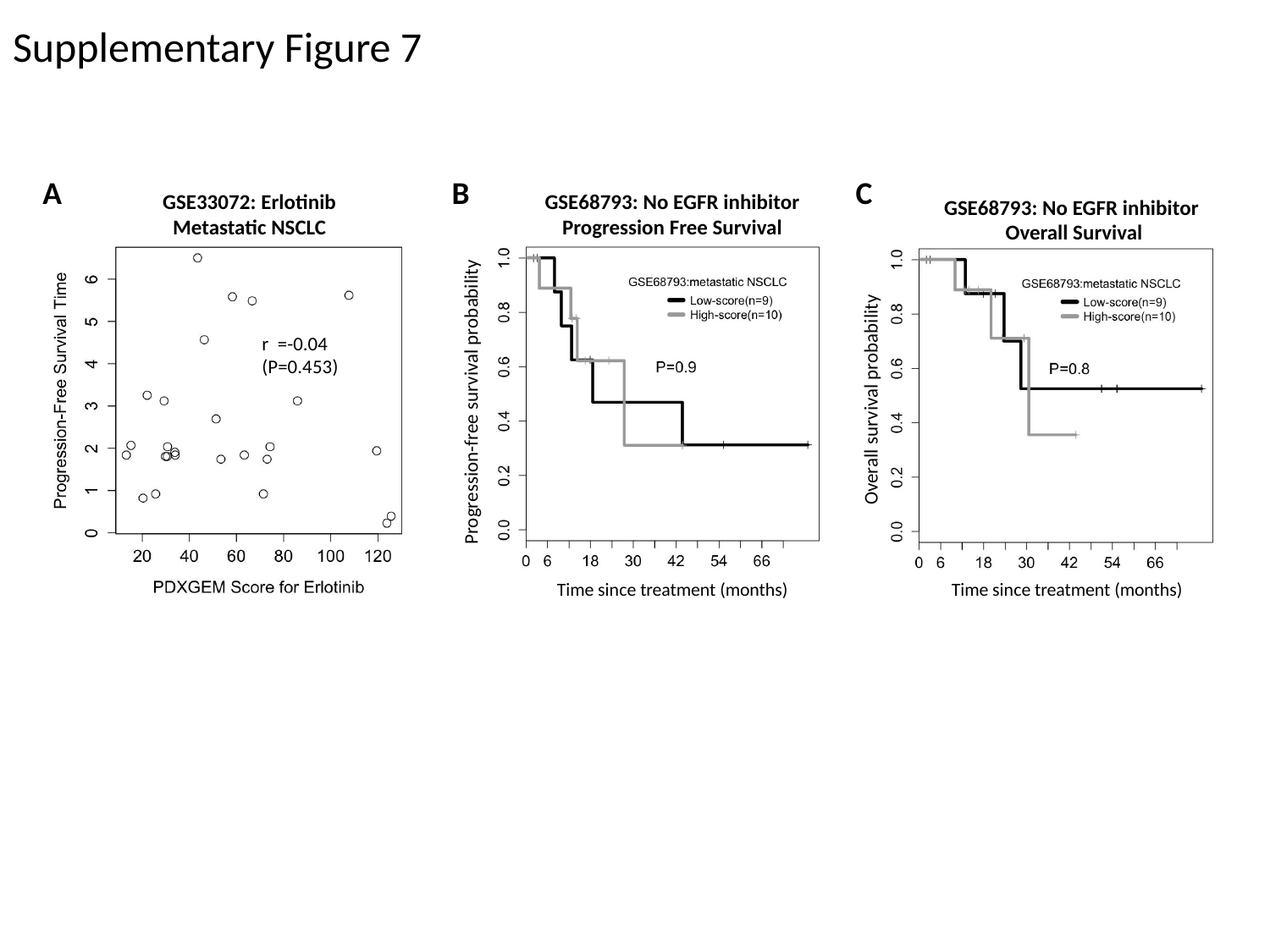

# Supplementary Figure 7
A
B
C
GSE33072: Erlotinib
Metastatic NSCLC
GSE68793: No EGFR inhibitor
Progression Free Survival
GSE68793: No EGFR inhibitor
Overall Survival
r =-0.04
(P=0.453)
Overall survival probability
Progression-free survival probability
Time since treatment (months)
Time since treatment (months)
