# Supplementary material for: PDXGEM: patient-derived tumor xenograft-based gene expression model for predicting clinical response to anticancer therapy in cancer patients
Source: BMC Bioinformatics. 2020 Jul 6;21:288. doi: 10.1186/s12859-020-03633-z (PMC7336455; doi:10.1186/s12859-020-03633-z)
Supplement: Supplementary file 3 — Additional file 3: Supplementary Table 2. File type: Word. The list of gene expression and anti-cancer drug response data sets [file 12859_2020_3633_MOESM3_ESM.docx]

**Supplementary Table 2.** The list of gene expression and anti-cancer drug response data sets

| **Cancer**  **type** | **GEO access ID**  **or website** | **Study type/ name** |  | **Gene Expression profiling**  **platform** | **Drug**  **response** | **Number of patients** | **Anti-cancer**  **Drugs** | **Role of data** |
| --- | --- | --- | --- | --- | --- | --- | --- | --- |
| PDX | GSE78806 | Novartis  PDX Panel |  | U133A | % change in tumor volume |  |  | Biomarker Discovery& Model Training set |
| Breast  Cancer | GSE3494  (Miller et al.) | retrospective tumor bank study |  | U133A |  | 251 | - | CCEA set |
|  | GSE20271  (Tabchy et al.) | Clinical trial |  | U133A | pCR | 91 TFAC | T+FAC or  FAC | validation |
|  | GSE22226  (Esserman et al.) | Clinical trial (I-SPY) |  | Agilent 4x44k | pCR | 120 TFAC |  | validation |
|  | GSE41998  (Horak et al.) | Clinical trial (NCT00455533) |  | U133A  2.0 | pCR | 127 | T+AC | validation |
|  | GSE25065  (Hatzis et al) | validation set in a prospective biomarker study |  | U133A | pCR | 498 | T+A |  |
|  | GSE42822  (Shen et al) | Clinical trial  (USO 02-103) |  | U133A | pCR | 91 TFAC | Trastuzumab + TFAC  or  TFAC alone | Validation |
|  | GSE32646  (Miyake et al) | ER-negative breast cancer study in Japan |  | U133+2 | pCR | 115 | TFEC | validation |
|  | GSE20194  (Shi et al.) | MAQC-II study |  | U133A | pCR | 278 | TFAC | validation |
| pancreatic ductal adenocarcinoma | GSE15471  (Badea et al) | Non-clinical trial |  | U133+2 |  | 78 |  | CCEA |
|  | GSE57495  (Chen et al.) | Non-clinical trial |  | Affymetrix  HuRSTA | OS | 63 | Gemcitabine | validation |
|  | E-MEXP-2780^*^  (Winter et al.) | Non-clinical trial |  | U133+2 | OS | 30 | N/A | Validation |
|  | ICGC | ICGC |  | RNASeq | OS | 96 | N/A | Validation |
|  | GSE17891  (Collison et al) | tumor bank study  at UCSF |  | U133+2 | OS |  | N/A | validation |
| Colorectal Cancer | GSE14095  (Watanabe et al.) | Non-clinical trial |  | U133+2 |  | 189 |  | CCEA |
|  | GSE62322  (Del et al.) | Non-clinical trial |  | U133A &U133B | pCR | 114 | FOLFIRI | validation |
|  | GSE39582  (Marisa et al.) | Non-clinical trial |  | U133+2 | OS | 585 | FOLFIRI or FOLFOX | validation |
|  | GSE5851 (Khambata-Ford et al.); | prospective randomized trial |  | U133A2.0 | pCR | 80 | Cetuximab monotherapy | Validation |
| Non-small cell lung cancer | GSE43580  (Tarca et al.) | Non-clinical trial |  | U133+2 |  | 150 |  | CCEA |
|  | GSE31625^$^  ^(Balko et al.)^ | Cancer cell line  Drug screening |  | U133A | Erlotinib-sensitivity | 18 / 28 | Erlotinib | validation |
|  | GSE37138  (Baty et al.) | Prospective clinical trial (SAKK 19/05) |  | HuEx-1.0 ST | pCR | 117 | Erlotinib+  Bevacizumab | validation |
|  | GSE33072  (Byers et al.) | Clinical trial  (BATTLE) |  | HuEx-1.0 ST | PFS |  | Erlotinib  at 2^nd^ line therapy | validation |
|  | GSE68793  (TCGA) | TCGA  Consortium study |  | U133A | PFS  OS | 135 | Non EGFR- inhibitors | validation |

**TFAC: paclitaxel, 5-FU, Adriamycin and Cyclophosphamide;
TFEC: paclitaxel, 5-FU, Epirubicin and Cyclophosphamide
FOLFIRI: folinic acid, 5FU, and irinotecan;
FOLFOX: folinic acid, 5F and oxaliplatin
pCR** : pathologic complete response, **OS** : overall survival, **PFS** : progression free survival
^*^ : Access ID of gene expression data at ArrayExpress
^$:^ Cancer cell line study
N/A: not available
